# The capsule of *Cryptococcus neoformans*

**DOI:** 10.1080/21505594.2018.1431087

**Published:** 2018-08-01

**Authors:** Arturo Casadevall, Carolina Coelho, Radames J. B. Cordero, Quigly Dragotakes, Eric Jung, Raghav Vij, Maggie P. Wear

**Affiliations:** Department of Molecular Microbiology and Immunology, Johns Hopkins Bloomberg School of Public Health, Baltimore, MD, USA

**Keywords:** cryptococcal capsule, polysaccharide structure, virulence factor

## Abstract

The capsule of *Cryptococcus neoformans* is its dominant virulence factor and plays a key role in the biology of this fungus. In this essay, we focus on the capsule as a cellular structure and note the limitations inherent in the current methodologies available for its study. Given that no single method can provide the structure of the capsule, our notions of what is the cryptococcal capsule must be arrived at by synthesizing information gathered from very different methodological approaches including microscopy, polysaccharide chemistry and physical chemistry of macromolecules. The emerging picture is one of a carefully regulated dynamic structure that is constantly rearranged as a response to environmental stimulation and cellular replication. In the environment, the capsule protects the fungus against desiccation and phagocytic predators. In animal hosts the capsule functions in both offensive and defensive modes, such that it interferes with immune responses while providing the fungal cell with a defensive shield that is both antiphagocytic and capable of absorbing microbicidal oxidative bursts from phagocytic cells. Finally, we delineate a set of unsolved problems in the cryptococcal capsule field that could provide fertile ground for future investigations.

The polysaccharide (PS) capsule of the human pathogenic fungus *Cryptococcus neoformans* is a beautiful structure, which appears in India Ink preparations as a translucent region (Figure 1). The capsule is the most important virulence factor of *C. neoformans*, contributing approximately 25% of the total virulence composite, as estimated from multivariate linear regression analysis to ascertain the contribution of different virulence factors [1], such that non-encapsulated mutants are avirulent [2,3]. Apart from its contribution to virulence, the capsule is important medically since its PS is the cryptococcal antigen used in diagnosis [4]. In recent years there have been several excellent reviews on the capsule that have focused on signaling stimuli and genetic regulation [5,6], immunological properties [7], role in interaction with phagocytic cells [8] and synthesis [9–12]. In this essay we will focus on the capsule as a cellular structure with the realization that even with this narrower emphasis the topic to be covered is still vast, which in combination with text limitations, necessitates a cursory discussion of the contributions of many investigators. In fact, our aim was to frame this essay to focus on the major problems in the capsule architecture field, without exhaustive detail with the goal of fostering discussion and experimentation to push the field forward. Furthermore, as indicated by the title, the reader is advised that the text below refers to *C. neoformans* unless it specifically mentions the closely related species *Cryptococcus gattii*.

**Figure 1.**
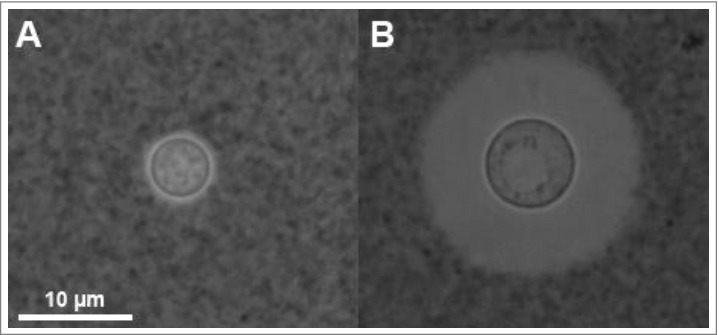
*Cryptococcus neoformans* yeast cells visualized by India Ink under phase contrast microscopy. India Ink particles are excluded from the dense PS capsule. A. Yeast cells cultured in an environment with optimal nutrition. B. Yeast cells cultured within mouse bone-marrow derived macrophages, experiencing starvation and oxidative stress from the phagolysosome for 24 h. Note the increased cell body, generation of what appears to be a large vacuole, and drastic increase in capsule size. Both images were obtained at 100x magnification with 2 × 2 binning.

The approach to the study of the capsule. The cryptococcal capsule is a notoriously difficult structure to study because it is primarily composed of water [13]. Consequently, any method that involves dehydration will damage its natural state. Imaging by electron microscopy requires sample dehydration, which results in clumping of PS molecules into thick strands that have unknown relevance to the native structure [14,15]. The PS dimensions measured by light scattering techniques are not consistent with the dimensions of PS fiber-like structures observed by EM [15]. Since the capsule is highly hydrated, this discrepancy is most likely due to the collapsing of adjacent capsule PS molecules following the fixation and dehydration steps required for EM analysis. The interconnectivity of PS fibers in the capsule after dehydration could reflect an artifact after loss of water and/or a manifestation of the high level of PS cross-linking in the capsule at native states. When observed by light microscopy, the capsule gives the impression of being a homogenous structure, however, it exhibits different densities in different regions such that the inner layer is denser, more rigid and less permeable to external solutes than the outer most layer [13,16–18]. India Ink penetration into equatorial regions of the capsule suggests radial differences in structure [19].

Our view of the capsule and its components is largely determined, as well as limited, by the method of isolation. For decades, much of our understanding of the capsule derived from the analysis of PS material recovered from the culture supernatants, based on the assumption that this material originated directly from the capsule via a still undescribed shedding process. However, once PS was directly isolated from the capsule and compared to secreted and capsular PS material it was apparent that these exhibited different physicochemical characteristics [20]. Also, the PS molecules are very large with masses ranging up to several million Daltons [21], which required shearing to explore PS structure by NMR. Solution light scattering analyses of PS preparations from various isolation methods demonstrated a wide range of dimensions with average molecular masses ranging from approximately 10^5^ – 10^8^ grams/mol, radii of gyration ranging from 150–500 nm and hydrodynamic radius raging 570–2343 depending on the method of isolation [20–23]. These macromolecular dimensions and their proportions are consistent with branched PS configurations [23]. PS branching and crosslinking of capsular and secreted PS are visible at the nanometer scale (30–100 nm) under various high-resolution microscopy techniques and preservations methods [15]. PS branching: 1) makes it possible for these polymers to reach very large dimensions; 2) explains the high level of crosslinking; 3) accounts for the porosity of the capsule to external solutes of <10 nm; and 4) produces interconnectivity of the capsule matrix that can explain its elastic properties and resistance to rupture with consecutive stretches [24].

Given their size and polydispersity, PS are very difficult to analyze since they are not amenable to crystallization and their size currently precludes a structure solution with NMR. Hence, when the knowledge problem regarding the capsule is considered as a function of the molecular mass of capsular components it is clear that we know most at the smallest and largest scales (Figure 2). The fragility of the capsule has motivated a search for alternative approaches to describe its hydrodynamic, mechanical and optical properties under conditions that minimize disruption of its native configuration, and from which inferences about macromolecular structure can be made [25,26]. Non-destructive methods to study the capsule include optical tweezers [27] and fluorescence microscopy with PS binding antibodies. Optical tweezers allow single-cell measurements of the mechanical or viscoelastic properties of the capsule [27]. These measurements exploit the strong interaction between the capsule and polystyrene beads, allowing the stretching of intact capsules and measure its stiffness or “Young's modulus” parameter from stress-strain curves. From these studies, we observed that the elastic properties of the capsule depend on several variables including cell age, the presence of divalent cations and whether antibodies have bound to the PS [24,27,28]. The ability of protective antibodies, but not non-protective antibodies, to increase capsule stiffness is also associated with interference with the budding process, suggesting another mechanism by which certain antibodies can mediate protection directly [28].

**Figure 2.**
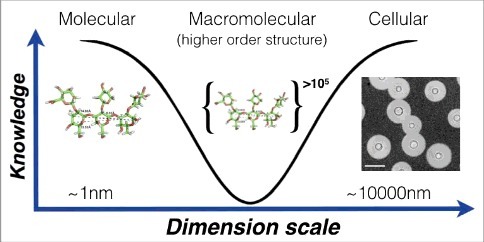
Knowledge of capsule structure as a function of dimension scale. Representative hyperbola of polysaccharide capsule structural understanding which is greatest at the smallest (molecular) and largest (cellular) levels. At the molecular level an *in silico* energy-minimized structure of the GXM M2 motif (see figure 3c) is estimated to cover a few nanometers in diameter. At the cellular level, capsule radii exist in the micrometer size range and are readily measured by negative staining and light microscopy (Scale bar in micrograph represents 10 µm). There is a knowledge gap in capsule structure at the macromolecular level or higher order structure (analogous to the secondary, tertiary and quaternary structural level of proteins) were many motifs are organized to form the overall capsular structure.

Since there are few methods to study the capsule directly without disturbing it, our understanding of capsular architecture must be a synthesis of information generated by various techniques. New insights into capsule structure may require new methods and technology. For example, a promising approach uses carbohydrate chemistry to synthesize oligosaccharides and study their structural and immunogenic properties [29,30]. Despite the difficulties involved in the study of the capsule, this area has been the object of intensive study for the past five decades and the information available allows us to synthesize a model for its architecture (Figure 3).

**Figure 3.**
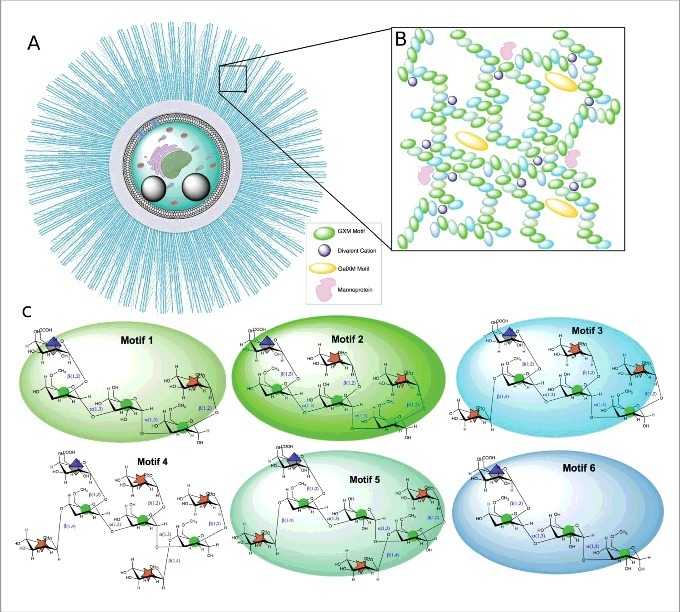
Illustration of the *C. neoformans* polysaccharide capsule structure. A. Cellular view of the polysaccharide capsule formed by a complex lattice-work of interconnected polymers that decreases in density, porosity and stiffness as it extends radially outward from the cell wall. B. Representative model of proposed polysaccharide lattice making up the capsular macromolecular structure involving interactions between GXM, GalXM, divalent cations, and mannoproteins. C. Structure of the six major motifs making up the GXM polysaccharide present in serotypes A, B, C, and D [35]. These tetrasaccharide to octasaccharide motifs fit together to make up the megadalton GXM polymer. Each motif is color coded to match the lattice illustration in B. In *C. neoformans* serotype A motif 4 is not present [35]. Motifs are represented using CFG nomenclature (mannose, green circles; xylose, orange 5-pointed start; glucuronic acid, blue/white diamond) along with chair pyranose rings and linkages.

The components of the capsule. The PS of the capsule are macromolecules of large dimensions cross-linked into a matrix that decreases in density, porosity and stiffness as it extends radially outward from the cell wall. The PS capsule has one major and two minor components. Glucuronoxylomannan (GXM) makes up ∼90% of the capsule and is formed by α(1,3) mannose backbone with ß(1,2) and ß(1,4) xylose and ß(1,2) glucuronic acid substitutions [31,32]. These structures occur in both *C. neoformans* and *C. gattii*, which differ in the degree of mannose backbone substitution. Structural differences in the PS give rise to antigenic differences that have been used to group cryptococcal strains into five serotypes known as A, B, C, D, and AD, with A, D and AD grouping with *C. neoformans* and B and C grouping with *C. gattii*. The mannose backbone can be O-acetylated on carbon six and this modification influences the serological activity, which reflects its importance in the chemistry and structure of the capsule [33,34]. Mannose O-acetylation also may affect the PS three-dimensional structure, which is particularly relevant to epitope identification and vaccine development. The density of this substitution in the overall PS remains unclear. Work by Cherniak and colleagues identified the repeating motifs of the GXM molecule; a glucuronic acid substitution is linked to every third mannose and different xylose substitutions producing a total of seven motif combinations (see figure 2) [35]. The glucuronic acid residues are negatively charged and their presence confer upon the capsule an anionic character. Glucuronic acids could also mediate non-covalent crosslinking of PS molecules via divalent cation bridges within the capsule, resulting in charge neutralization of the GXM molecule and capsule size increase [36]. A single molecule of GXM may include different motif combinations, demonstrating the complex heteropolymeric structure of GXM [37]. A secreted lactonohydrolase of *C. neoformans*, LHC1, was recently implicated in the formation in capsular higher order structure suggesting enzymatic remodeling after PS assembly in the extracellular space [38].

The two minor components of the capsule are GalXM and mannoproteins, of which the latter are highly diverse and immunogenic [39]. In addition to the PS components the capsule contains lipid structures whose function is unknown [40, 41]. Recent work has shown that GalXM is more correctly referred as GXMGal [42] but we will use the older nomenclature to maintain the continuity of the literature. It is unclear if GalXM is a true component of the capsule or an export polysaccharide but all three components occupy spatially different regions of the capsule [43]. The study of GalXM is made easier by the cap67 deletion mutant which produces only GalXM and not GXM [44]. The α(1,6) galactan backbone of GalXM is substituted at every second residue with α(1,3) manose, ß(1,4) galactose side chains which are additionally substitute with ß(1,2) and ß(1,3) xylose and ß(1,3) glucuronic acid residues [35,42]. Utilizing 1D and 2D NMR, Previato and colleagues have been able to show the location of the O-acetylation and ß-galactofuranose substitutions in two populations – GXMGal and NGalXM – of GalXM [45]. While research has identified the molecular composition and primary structure of both GXM and GXMGal, how these monosaccharides come together to form three-dimensional structure of >1 mDa PS capsule remains to be solved [5] (Figure 3). Recent work in the bacterial PS field indicates that with the use of specific HPLC isolation techniques and new 2D NMR techniques the three-dimensional structure of capsular PSs can be determined [46,47], and these methods may be applicable to *C. neoformans* PS.

The Dynamics of the Capsule. The exact mechanism of capsule growth remains elusive. Capsule sizes vary within clonal populations and between cryptococcal species, and size is affected by culture conditions [48] . Analysis of PS from cells with large and small capsules suggests that capsular enlargement occurs by generating subunits with larger diameters to expand upon the existing capsule [22]. There is evidence that capsule growth also involves the intermixing of old and new capsule subunits with newer subunits closer to the outer edge of the capsule [49]. Culturing cryptococcal cells under alkaline conditions encourages capsule growth, suggesting a potential role for ionization states of the PS subunits acidic side groups [48]. Capsule size is also regulated by the cell cycle [50] and is larger in cells grown in media conditions with slower growth rates [48].

*C. neoformans* reproduction by budding must necessarily involve local capsular rearrangements. Although this process is complex and depends on the size of the capsule in the mother cell, there is evidence that when cells bud the capsule of the parent cell collapses inward generating an invagination through which the bud is expelled [49]. This invagination creates a tunnel through which the bud can exit while keeping its own capsule intact and separate from the mother [49]. Cryptococcal capsule growth appears to be coordinated with the cell body growth and replication, such that it enlarges primarily during the G1 phase while its growth arrests during the budding process [50].

The capsule is a highly dynamic structure. Soon after infection in lung tissues, the capsule dramatically increases in size [51]. *In vitro*, the capsule can grow as fast as 0.3–2.5 umˆ3/min and the rate of its growth appears to influence its final size [52]. The capsule size and structure reflects the extracellular environment. Different from the large capsule sizes in yeast cells isolated from lung tissue, brain samples show small capsule sizes [53]. Conditions stimulating capsule enlargement *in vitro* include: low iron [54], high CO_2_ [55], mammalian serum [48], mannitol [56] and nutrient starvation. Small capsules are observed under high osmotic pressure [57], high iron and nutrients. While capsule growth seems to be an irreversible process [49], there is some evidence for capsule reduction during aging [24]. Capsule size is not the only feature that can change and once it reaches a final size it continues to change. The porosity and density of the capsule varies in different conditions [16,17,26,53]. *C. neoformans* cells isolated from infected tissues exhibit capsules with higher density and lower porosity based on the diffusion of external solutes of defined diameter [17]. Based on the reactivity and binding pattern of monoclonal antibodies, the antigenic structure of the capsule also changes depending on the tissue and the age of the cell [19,53,58]. Capsules from older cells exhibit higher rigidity, decrease permeability, electrometric potential and composition [24]. Considering that the capsule is the first microbial component to interact with the immune system, its dynamic nature could contribute to virulence by evading immune defenses in already immunocompromised hosts.

The Function of the Capsule in the Environment. PS capsules are known to protect microbes from desiccation [59] and although this has not been shown for *C. neoformans* it is likely to apply to this organism. Similarly, the cryptococcal capsule protects from oxidative stresses and increases in capsule size result in stronger protection [60]. In addition, the capsule can be used to defend against amoeba, a natural predator of environmental yeasts [61]. This is supported by evidence showing that interactions with amoeba result in larger capsule sizes [62]. Additionally, if the yeast is phagocytosed the PS capsule has been shown to confer resistance to oxidative stress, a significant component of phagolysosomal degradation [57] .

The contribution of the capsule to virulence. The capsule of *C. neoformans* contributes to virulence through several mechanisms that synergize to confer protection to yeast cells against host phagocytic cells and interfere with host immune mechanisms. The capsule is antiphagocytic such that there is essentially no ingestion of yeast cells in the absence of antibody- or complement-derived opsonins *in vitro* [63]. The capsular PS is poorly immunogenic and seldom elicits strong IgG responses such that opsonic antibodies are either scarce or absent [64] and cryptococcal infection is associated with complement depletion [65]. The epitope recognized by protective Abs is unknown, except for the fact that acetylation of GXM is critical for recognition and deacetylation leads to loss of Ab binding. A small number of phagocytic events may derive from low titers of circulating antibodies generated by the adaptive immune response, or alternatively, opsonization by naturally occurring IgM [66]. While complement system opsonins are readily available in serum, the efficacy of this opsonin varies with the strain. When deposited near the capsule surface complement is an effective opsonin but in some strains it is deposited deep inside the capsule, which precludes receptor engagement [67]. When opsonic antibody is available, combined with complement binding to the surface of the capsule, significant ingestion of yeast cells by macrophages occurs [67,68]. *Cryptococcus* spp. are remarkable for establishing chronic infections, often lasting months to years [69]. During that time, the yeast cells in tissue are subject to selection by host defense mechanisms. Analysis of sequential isolates from individuals who are chronically infected reveals changes to the capsular PS [70] and its production [71], consistent with strain microevolutions. Aged cells exhibit numerous changes relative to younger cells that could contribute to virulence and persistence in tissues [72]. Such changes could help *C. neoformans* persist in tissue.

Capsular PSs have powerful immunomodulatory properties (reviewed in [7]). GXM and GalXM have different immunomodulatory properties [73], which may act synergistically to degrade immune function. In fact, when PS preparations are fractionated by size the different components differ in their ability to stimulate cytokine responses [74]. The immunomodulatory properties of these PS have shown encouraging result in experimental models of immunotherapy with encouraging results in rheumatoid arthritis [75] and endotoxin shock [76]. GXM can bind to TLR2/4 and CD14 [77] and at least CD14 has been shown to mediate ingestion of *C. neoformans* by swine microglia [78]. However, the contribution of engaging these receptors is unclear since neither TLR2/4 or CD14 deletion significantly affects mouse survival upon *C. neoformans* challenge. The biophysical properties of extracted capsular PS correlated with their ability to influence ingestion and resist host oxidative attack [23]. However, viscosity of *ex vivo* capsular PS was not associated with virulence [79].

The relationship between strain capsule size and virulence, if any, remains uncertain since different studies have produced conflicting results. Zebrafish infected with *C. neoformans* strains with larger capsules had worse survival [80]. Other studies report that infection with hypocapsular strains [81,82] correlates with worse clinical outcomes, while in yet another study the presence of isolates with larger capsules correlated with higher intracranial pressure [79]. Critically, the same authors found that capsule size *in vitro* did not correlate with capsule size in patient's brain [79]. However, a mouse study found that both hypercapsular and hypocapsular strains were less virulent for the mouse [83]. It may be possible to reconcile the divergent observations if one considers different responses in each host tissue as well as strain-specific differences in capsule size. In this regard, a single strain can manifest differences in capsule size depending on the infected organ [84]. It is possible that a large capsule is beneficial for yeast survival in the lung environment but impedes dissemination to the brain. Isolates with smaller capsules invade the brain more efficiently and the consequences of brain proliferation are much more severe to the host, with a net result of increased mortality for hypocapsular strains [83]. An alternative explanation is that capsule size reflects starvation of *C. neoformans*, hence a smaller capsule indicates a yeast that is not starved and is well adapted to host survival, hence capable of causing higher host mortality. While other functions of capsule-regulating genes on the overall virulence of the strain must be considered, size alone may not be an appropriate biomarker for virulence. Overall, the presence of a capsule is critical for *C. neoformans* survival and virulence in the host; differences in outcome depending on capsule size could reflect specific experimental condition.

Capsule as a drug target and role in drug resistance. The requirement for the capsule in cryptococcal virulence suggests that interference with PS synthesis is a potential anticryptococcal therapeutic strategy. In this regard, the finding that mAbs to the capsule slowly hydrolyze the PS suggests that some of their protective functions could involve direct effects on the capsule [85]. Although not fungal specific, the inhibitors of the electron respiratory chain, salicylhydroxamic acid and antimycin A [86], as well as sodium butyrate, can inhibit capsule growth [87]. Amphotericin B treated cells manifested smaller capsule suggesting that some of its antifungal effects *in vivo* could be due to rendering cryptococcal cells more susceptible to host defense mechanisms [88]. There is some evidence that the capsule protects *C. neoformans* against polyenes [89]. Cryptococcal cells with larger capsules showed resistance to amphotericin B while acapsular mutant, Cap59, was more susceptible [60]. The PS capsule is thought to prevent the uptake of this bulky antifungal molecule. Similarly, the presence of the capsule protected *C. neoformans* against glycolipid hydrolyze inhibitors [90]. Interestingly, the opposite effect was observed with fluconazole, whereby acapsular mutants were more resistant than encapsulated wildtype [60]. The proposed mechanism for this unusual result is due to the hydrophilicity of fluconazole aiding in the uptake through the hydrophilic PS capsule. However, capsule enlargement was also associated with increased resistance to fluconazole [91].

## Unsolved problems

We end by posing several questions relating to capsule architecture that remain unanswered. Although we do not mean to insinuate that these are the only unsolved questions, or even the most interesting, they piqued our interest while reviewing the material summarized above.

1. How are monosaccharides assembled into macromolecules that can reach micrometers in lengths? In this regard, PS capsule size is regulated at the polymer level [92] and the capsule can reach many micrometers in radius by polymerizing monosaccharides into a still unknown conformation.
2. What are the mechanisms that regulate capsule size? At some point the capsule stops growing and we still do not know how the cell knows when to halt this process. Some studies have suggested that capsule size limit must be influenced by the surface area of the cell body [49], the rate of its growth [50], or nutrient status [48].
3. What are the mechanisms by which the capsule is rearranged during budding? Is there an active process to move the PS fibrils and create channels for the emergence of buds in large cells?
4. What portions of the capsule are synthesized intracellularly and extracellularly? Present views hold that capsular PS is synthesized in vesicles intracellularly that are then exported to the extracellular space [93,94]. However, we don't know whether vesicles simply export the building blocks or the assembled PS molecules. It is important to note that mutations in the *C. neoformans* secretory system still exhibit some capsule suggesting a capsule assembly process independent of vesicle transport [95–97]. In this regard, it is possible that *C. neoformans* also uses a capsule synthesis process analogous to bacterial capsule which are synthesize at the cell surface.
5. What is the role of lipids in capsular structure? Lipid structures have been observed by electron microscopy and lipid staining [41,98] and intracellular PS synthesis appears to be closely associated with lipid structures [99] . The origin, location and purpose of capsule lipids and their role in capsule structure and synthesis is not well understood.
6. What is the relationship between the capsule and the shed PS? Is the shed PS material sloughed off from capsule occurring during capsular rearrangements or material made for export? How is the capsule attached to the cellular surface? *C. neoformans* PS has been shown to bind the cell surface in a process that can involve direct interactions with glucans [100] and chitin oligomers [101]. However, it is unclear whether these interactions are comparable between polysaccharides added to acapsular cells and wild-type capsular synthesis.
7. How are the PS molecules assembled into capsule? Is there a fractal dimension to the PS capsule and what is it? What is the secondary structure of GXM and GXMGal? What is the chemistry of the branching structures?
